# A Geographical Heuristic Routing Protocol for VANETs

**DOI:** 10.3390/s16101567

**Published:** 2016-09-23

**Authors:** Luis Urquiza-Aguiar, Carolina Tripp-Barba, Mónica Aguilar Igartua

**Affiliations:** 1 Department of Network Engineering, Universitat Politècnica de Catalunya (UPC), C. Jordi Girona 1-3, Barcelona 08034, Spain; luis.urquiza@entel.upc.edu; 2 Departamento de Electrónica, Telecomunicaciones y Redes de Información, Facultad de Eléctrica y Electrónica, Escuela Politécnica Nacional (EPN), C. Ladrón de Guevara E11-253, Quito P.O.Box 17-01-2759, Ecuador; 3 Faculty of Informatics, Universidad Autónoma de Sinaloa (UAS), De los Deportes Avenue and Leonismo Internacional, Mazatlán 82107, Mexico; ctripp@uas.edu.mx

**Keywords:** heuristic optimization, routing protocols, VANET

## Abstract

Vehicular ad hoc networks (VANETs) leverage the communication system of Intelligent Transportation Systems (ITS). Recently, Delay-Tolerant Network (DTN) routing protocols have increased their popularity among the research community for being used in non-safety VANET applications and services like traffic reporting. Vehicular DTN protocols use geographical and local information to make forwarding decisions. However, current proposals only consider the selection of the best candidate based on a local-search. In this paper, we propose a generic Geographical Heuristic Routing (GHR) protocol that can be applied to any DTN geographical routing protocol that makes forwarding decisions hop by hop. GHR includes in its operation adaptations simulated annealing and Tabu-search meta-heuristics, which have largely been used to improve local-search results in discrete optimization. We include a complete performance evaluation of GHR in a multi-hop VANET simulation scenario for a reporting service. Our study analyzes all of the meaningful configurations of GHR and offers a statistical analysis of our findings by means of MANOVA tests. Our results indicate that the use of a Tabu list contributes to improving the packet delivery ratio by around 5% to 10%. Moreover, if Tabu is used, then the simulated annealing routing strategy gets a better performance than the selection of the best node used with carry and forwarding (default operation).

## 1. Introduction

Vehicular ad hoc networks (VANETs) [1] are a special case of mobile ad hoc networks (MANETs), where nodes are vehicles that interchange data to establish and maintain communication. Vehicular applications can be divided into safety, vehicular traffic efficiency and infotainment applications [2]. Traffic flow control or environmental conditions monitoring are some aims of such applications. The efficiency-oriented applications require a continuous monitoring phase of the streets and city conditions. Vehicles can gather this information and feed the monitoring centers through the VANET. Vehicles can reach the watching center using Vehicle-to-Infrastructure (V2I) or Vehicle-to-Vehicle (V2V) communications, in a direct or multi-hop fashion, respectively. Challenges in VANETs, such as fast topology changes, low link lifetime or a potentially high number of nodes taking part in the network, have encouraged researches to propose geographical routing protocols for multi-hop communication in VANETs as an alternative to the classical topology-based routing approach. Geographical protocols, also known as position-based protocols, make their routing decision using local information, mainly nodes’ positions.

Two procedures can be recognized in the operation of geographical routing protocols: the forwarding mechanism, which defines the rules that a node follows to choose the next forwarding hop; and the recovery strategy, used by a node when it does not find any neighbor that meets the forwarding criteria.

One of the first and widely used classifications of VANET routing protocols presented in [3] distinguishes two types of recovery strategy: the so-called “carry and forwarding” that consists of storing a packet until the node finds a suitable next forwarding node. This “carry and forwarding” strategy introduces in general high delay in data transmission. Hence, it is adequate for applications for Delay-Tolerant Networks (DTN), which are the target applications in this paper. The second recovery method is for Non-DTN protocols. There are two most common alternatives: (1) the use of the right hand rule; and (2) the construction of a recovery path through request/reply signaling messages. As the DTN recovery approach has increased its use in VANET routing protocols, a recent classification [4] of VDTN (Vehicular Delay-Tolerant Network) uses geographical knowledge needed by the routing protocols to differentiate them. This information can range from only the necessity of get the geographic location of nodes, to use road maps or even to use on-line information.

Regarding the forwarding mechanisms in geographical routing, there are some classifications depending on the factor used to differentiate them. According to [3], geographical protocols can be classified in non-overlay, in which all of the nodes of the network make routing decisions; and overlay, if only some nodes of the network are allowed to change the routing decision of a packet. A recent classification of geographical routing protocols that consider traffic and network status in their routing metrics [5] identifies protocols that construct the full path using information like distance between nodes and vehicles’ density, among others, which is not the common approach. Other protocols make routing decisions in junctions or the anchor or in each node.

Updated surveys of geographical routing protocols [5,6,7] show that the forwarding criteria of these protocols have evolved from considering only the geographical distance between nodes as in Greedy Perimeter Stateless Routing (GPSR) [8] to other routing proposals that require detailed geographical information and include other additional metrics, like the speed and direction of the vehicle. Nevertheless, despite the enhancements in the routing decisions, the selection of the best node as the next hop maintains as predominant in the routing criterion among most of the geographical routing protocols.

In [9], the authors show that any geographical routing protocol that operates under a hop-by-hop forwarding strategy can be understood as a direct application of the general “local search” algorithm of discrete optimization. Furthermore, they propose to adapt some other heuristic to geographical routing protocols for VANETs in order to improve the results obtained with local-search algorithms.

In this work, we propose a generic Geographical Heuristic Routing (GHR) protocol that can be applied to any DTN geographical routing protocol that makes forwarding decisions at each hop. Our proposal combines all of the adaptations presented in [9] for the forwarding and recovery phases of the protocol, which are based on Tabu search and simulated annealing.

Our paper offers a thorough performance evaluation of GHR with MMMR, a traffic-aware routing protocol suitable for delay-tolerant applications. Our study analyzes all of the meaningful combinations that our generic algorithm provides. Finally, to provide a complete analysis of our findings, we assess MANOVA and paired statistical *t*-tests to identify which performance differences are significant. This analysis takes into account the effect of the vehicle density in the appearance of differences. We found that some features of GHRbehave better than others depending on the vehicle densities or the applications’ requirements.

The rest of the paper is organized as follows: Section 2 summarizes some other works that use optimization techniques in wireless networks. Then, Section 3 present the Tabu search implementation used in our proposal, in addition to the simulated annealing strategy and a generic routing procedure. Section 4 describes the algorithm of our heuristic routing protocol proposal. Next, Section 5 is devoted to the performance evaluation of our contribution, which includes the description of the simulation scenario and the statistical analysis of the simulation results. Finally, conclusions and future work are drawn in Section 6.

## 2. Related Work

Heuristic optimization techniques have been used in some works to improve the operation of routing protocols. In [10], the authors propose a new routing protocol called the Tabu Search-based Routing Protocol (TSRP), which introduces an implementation of the Tabu search meta-heuristic to avoid nodes previously selected in the routing process of the data from the sensor (that has previously sensed the event) to the sink. TSRP is based on maximizing the cost function, which considers the energy and the visibility of that sensor compared to the sink. TSRP keeps a 0/1 string that indicates whether a node forwarded a packet or not. Simulation results show that TSRP extends the network lifetime compared to the protocols Gossiping [11] and MFR [12].

The authors in [13] propose a routing optimization algorithm to minimize the route cost from a source to a destination within a reasonable time. The proposed algorithm is designed by using a tabu search mechanism that is a representative meta-heuristic algorithm. The tabu search algorithm carries out two neighborhood-generating operations in order to determine an optimal path and minimize algorithm execution time. The proposed tabu search algorithm is compared with other meta-heuristic algorithms, which are the genetic algorithm and simulated annealing, in terms of the routing cost and the algorithm execution time. The comparison results show that the proposed tabu search algorithm outperforms the other algorithms and that it is suitable for adapting the routing optimization problem.

The optimal parameter setting of the Optimized Link State Routing (OLSR) is analyzed in [14], also as an optimization of routing in ad hoc networks. The authors use a series of representative meta-heuristic algorithms (particle swarm optimization, differential evolution, genetic algorithm and simulated annealing) in order to find automatically optimal configurations of this routing protocol. This study is focused on VANETs and is tested with realistic scenarios based on the city of Malaga. In the experiments, the evaluated OLSR configurations obtain better Quality of Service (QoS) than the standard RFC 3626 [15].

Position-based routing relies on Global Position System (GPS) coordinates. In [16], the authors focus the study on the fact that the GPS measured data will always be corrupted by noise and other factors. The authors use the simulated annealing optimization technique for finding lower bounds on how much improvement is possible given the inaccuracies in the measurements. They use two algorithm previously proposed, VLOCI [17] and VLOCI2 [18], in order to improve the initial GPS coordinates.

In general, there are several routing protocols proposed for wireless mobile ad hoc networks. Particularly in vehicular networks, depending on the particular scenario, one protocol or another might lead to a better result. However, we have not found any routing protocols that use other heuristic optimization techniques in the routing decision process apart from the greedy approach of geographical routing protocols. All of the position-based protocols surveyed in [3,5,6,7,19] work with local search and follow a greedy approach in the selection step of the next forwarding node. Hence, there is room for performance improvement of geographical protocols by using other heuristic optimization techniques like Tabu search and simulated annealing.

## 3. Background

This section presents the algorithm for a generic DTN geographical routing protocol and summarizes the suggested implementation of Tabu search and simulated annealing for the geographical routing proposed in [9].

### 3.1. Generic Geographical Routing Process

Algorithm 1 shows the generic routing process of a packet in a node that employs a geographical routing protocol with carry and forwarding as the recovery strategy. The algorithm is written for an anycast routing protocol. Nonetheless, the algorithm works exactly the same for a unicast routing. In that particular case, the destination set Dst used as the input of the algorithm will have a single element that is the destination node of the packet.

**Algorithm 1** General routing process of a DTN geographical protocol.Route(*P*)**Require:** a packet *P*, Destination set *Dst*, current vehicle *v***Ensure:** Forward *P* to a vehicle neighbor *v*_nh_ to reach a member *Dst* or save *P* in buffer.1:Dst←GetDestinationSet(*P*)2:**if** v∈Dest **then**3: GotoUpperLayer(*P*)4: **return**5:**if** ∃n∈ N(*v*) ∧n∈Dest∧ IsLegal(n)=True **then**6: $v_{\mathrm{nh}}\leftarrow n$7:**else**8: $v_{\mathrm{nh}}\leftarrow$ DoRouting(P,v,Dst)9: **if** $v_{\mathrm{nh}}\neq v$ **then**10:  Forward($P,v_{\mathrm{nh}}$)11: **else**12:  Buffering(P)13:**return**

When a packet arrives to a node, the first step is to determine the destination set of that packet (Line 1 in Algorithm 1). The information of the destination set can be retrieved from the anycast IP address of the packet. A list of destination nodes is associated with each anycast IP address. Every node carries this mapping table with it. Therefore, the look-up task of the destination set does not need any on-line search. Moreover, since the typical destination set will be fixed network points like RSUs, then their positions also can be a priori known. The associations anycast IP address-destination and RSU position do not need to be constantly updated. A vehicle can refresh this information where there are available Internet connections, for instance when the vehicles are parked at home or at the office.

If the current node *v* is a member of the destination set Dst, then the packet is processed by the upper layer (Line 3 in Algorithm 1), and the routing process ends. When the current node is not a destination member, it searches if it has a member *n* of the destination set among its neighbors N(*v*), which in addition is considered a legal node (see Line 5). A “legal node” refers to a neighbor that meets a certain list of characteristics that depend on the routing protocol. For instance, in the case of our MMMR [20] protocol used in this paper, a legal neighbor has to be in LOS, and the estimated power reception has to be higher than its antenna sensibility plus an extra margin of 1 dB. If the current node *v* finds such a neighbor, then it chooses as the next forwarding node that neighbor *n* (Line 6). Otherwise, a selection of the next forwarding hop $v_{\mathrm{nh}}$ is performed (Line 8). Next, if the selected node $v_{\mathrm{nh}}$ is not the current node *v* (Line 9 in Algorithm 1), then the routing protocol forwards the packet to $v_{\mathrm{nh}}$. Else, the packet is stored in the buffer until a suitable node appears. The next sections explain in detail two routing proposals for the function DoRouting, which is in charge of selecting the next forwarding node.

### 3.2. Heuristics Adaptations for VANET Routing

Local search with greedy selection is the predominant strategy in geographical VANET routing [3,5,6,7,19]. Since this mechanism has obtained good results, it is important to test other heuristics, like Tabu search and simulated annealing adaptations to the VANET routing. Tabu search and simulated annealing are two meta-heuristic widely used in combination with local search that obtain good solutions in too complex and big discrete optimization problems. Adaptations of both of them have been proposed in [9] to operate in multi-hop VANET routing. In this section, we summarize these adaptations.

#### 3.2.1. Tabu Search

In [9], the Tabu search implementation was proposed as a list called tabu *τ* of the last *k* nodes visited by a packet. The key idea is to keep track of the nodes already visited and forbid forwarding to the nodes included in the tabu list *τ*. The tabu list *τ* is updated following the logic of a FIFO list. This means that the oldest visited node in the list is the first to be deleted if the list gets full. Using the tabu technique, we are able to avoid loops. Nodes will be included in the list of forbidden nodes if they are not solutions (destination nodes).

Contrary to the implementation of [10] that uses one bit for each node of the network in the tabu list, in our tabu search, every packet carries the entire ID of a limited number of nodes in the tabu list *τ* (the last ones, e.g., the last three ones). The number of nodes in a VANET is very dynamic and relatively big, so it is not practical nor possible to use a 0/1 string to encode all of the nodes of a VANET. Moreover, our tabu list implementation of a packet is a dynamic, temporal filter to determine legal nodes in addition to the statical requirements established by the routing protocol. This dynamic filter forbids using a node in the tabu list as a next hop for that packet during a limited number of forwarding operations (the length of the list). For instance, if the length of the tabu list is equal to four, then a node added to the tabu list can be used for routing the packet again only after four routing operations from its addition to the list.

**Figure 1:**
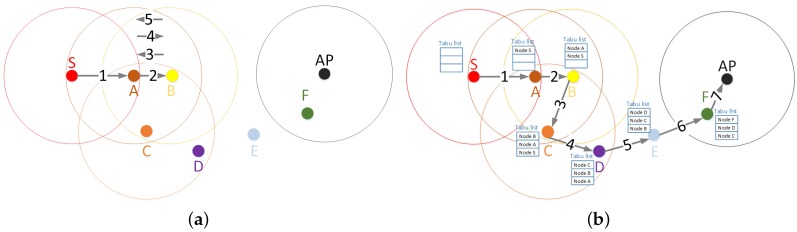
An example that shows how Tabu avoids loops and improves the operation of a basic routing protocol based on forwarding the packet to the closest neighbor to destination. (**a**) Forwarding to the closest neighbor fails when two nodes are both the closest neighbors to destination, as Nodes A and B in this topology; (**b**) Tabu breaks the loop between Nodes A and B. Tabu list prevents B from returning the packet to A. Node C receives the packet from B.

To show how Tabu could enhance the operation of a routing protocol, we assume in our example, depicted in Figure 1, that nodes employ a basic routing procedure, in which nodes always forward the packet to the closest neighbor to destination, even when the best neighbor is farther than the current forwarding node to the destination.

On the one hand, our simple routing protocol will fail in the packet delivery between S and AP, as can be seen in Figure 1a, because there is a loop between Nodes A and B. The problem occurs because the closest neighbor to the destination from Node B is the previous hop of the packet (i.e., Node A). Therefore, Node B will return the packet to Node A, and then, Node A will forward the packet to Node B again, and so on. This creates a loop between Nodes A and B because both are the closest nodes to the destination.

On the other hand, if the routing protocol implements a tabu list to record the previous hops, then the packet transmission from S to AP could overcome the loop between A and B in the topology of our example. In this case, depicted in Figure 1b, when Node B receives the packet from Node A, B cannot forward it again to A (its closest neighbor to the destination) because A is contained in the Tabu list of the packet. Therefore, Node B has to forward the packet to C. From Node C, the packet finds a path to reach the destination. In conclusion, Tabu provides memory to the routing decision and helps nodes to deal with complex network topologies in order to find routing paths.

#### 3.2.2. Simulated Annealing

The Simulated Annealing (SA) adaptation to VANET routing protocols [9] aims to provide a controlled and decreasing randomness in the forwarding process. The randomness in the forwarding decreases when a packet approaches the destination. To do that, simulated annealing employs Equation (1). If there are closer neighbors to the destination with respect to the current node (forwarding phase), this equation provides the probability of selecting a random legal node as the next forwarding hop. Otherwise, in the recovery phase in which there is not a closer node to the destination, Equation (1) represents the probability of choosing a node farther than the current node. The idea behind selecting a worse node than the current one in terms of distance is to avoid buffering and to consider other alternative forwarding nodes.

(1) $$p=e^{-\gamma \frac{|d_{s-s^{\prime }}|}{d_{s}}}$$

The metrics used to compute the probability *p* of Equation (1) are:

- The distance of the current node to destination $d_{s}$. As a packet jumps, it should be approaching to the destination, and $d_{s}$ should therefore decrease. This should tend to reduce the probability *p*.
- $|d_{s^{\prime }-s}|$ is the absolute value of the difference of distances between the best forwarding candidate to the destination (named $d_{s^{\prime }}$) and the current node to destination $d_{s}$. If $d_{s^{\prime }-s}$ is high, then it will contribute to reducing probability *p*. The idea of the numerator is that if the next position of the packet by choosing the closest forwarding node is not far from the current position (i.e., the best candidate improves little in distance), then it could be a good option to choose randomly and try other paths that otherwise would not have been explored because of the benefit of using the greedy selection, i.e., choosing the best candidate.
- *γ* is a tuning parameter to balance the deterministic and randomness behavior. If it is close to zero, the probability *p* tends to one. Conversely, if γ→∞, then p→0.

**Figure 2:**
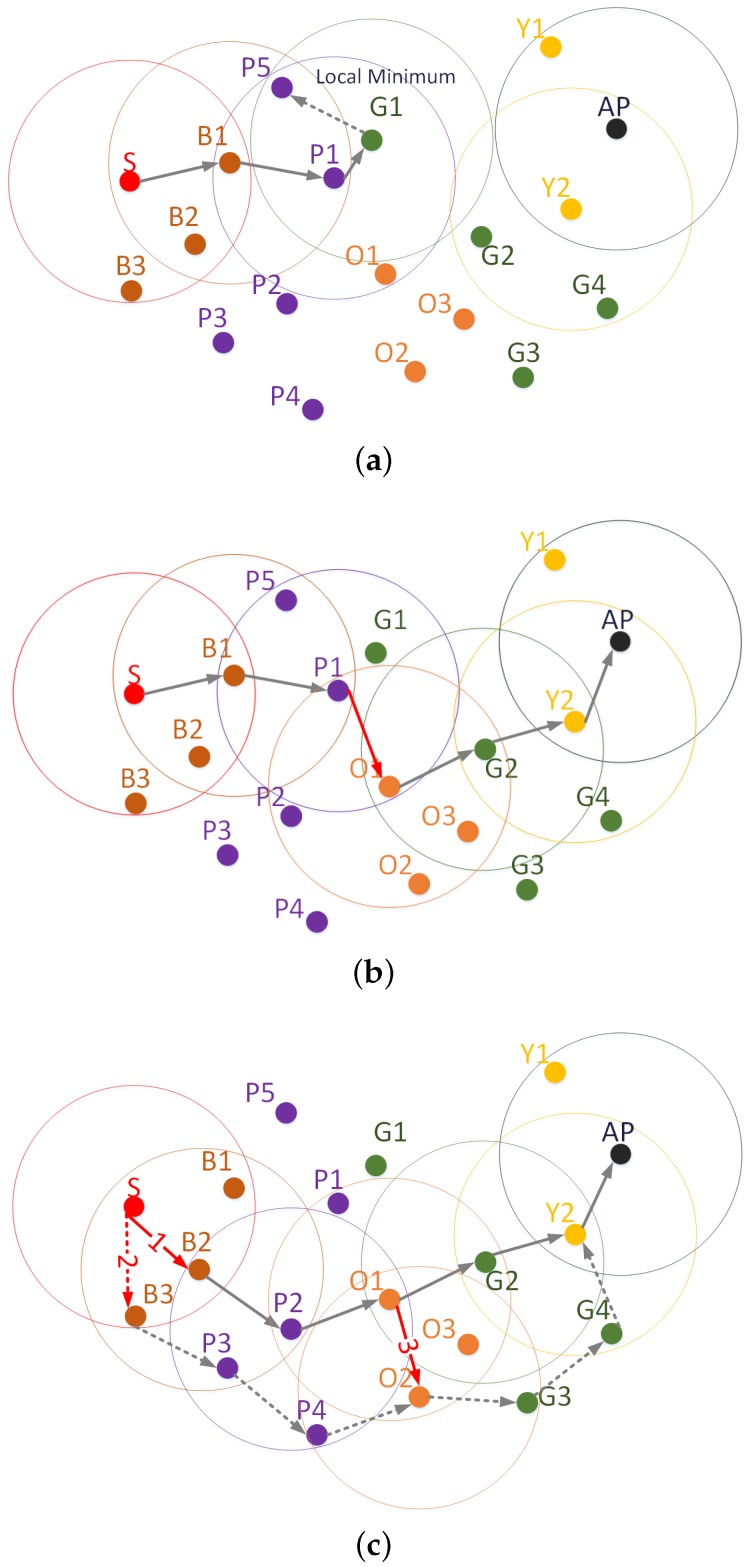
An example that shows how Simulated Annealing (SA) could help routing protocols avoid local minimum scenarios. (**a**) Node G1 (or P5 if Tabu is used) is the last hop in the path between S and AP. The path S to G1 (or P5) was built using greedy forwarding. Greedy forwarding can lead to “local minimums”. (**b**) There is a high probability *p* to forward the packet randomly, according to the SA principle, when it arrives at P1 because the improvement in the distance (numerator in Equation (1)) by selecting G1 is little. (**c**) There is a high probability *p* to forward the packet randomly at the beginning of the path from S to AP because the distance to the destination is long (denominator in Equation (1)).

The advantage of simulated annealing can be seen in a topology as the one depicted in Figure 2. In that topology, a greedy algorithm that always forwards packets to the closest node to the destination will fail in the delivery of packets from S to AP. Such a kind of protocol will follow the path shown in Figure 2a. That greedy path will end in the node G1 or, if Tabu was used, in the node P5. In any case, the routing algorithm will not find any suitable next hop.

On the contrary, if simulated annealing is considered in the strategy of the routing protocols, then other different scenarios are possible. Figure 2b shows one of them. In that scenario, the P1 node decides to forward the packet randomly and chooses the node O1 (red link in the path of Figure 2b). The reason for the random forwarding decision of P1 is the short distance improved by its best neighbor (Node G1) with respect to its distance to destination (P1). This short difference of distance is precisely the numerator of the fraction used by the simulated annealing equation to compute the probability of random forwarding and makes the value of *p* high. Once O1 receives the packet, the most likely routing behavior is to forward the packet following the greedy (deterministic) approach. This is because the other nodes (i.e., G2 and Y2) improve the distance to the destination at each hop considerably with respect to the previous node in the path, and the total distance to the destination is reduced, as well. Therefore, the random forwarding probability, which depends on these two factors, becomes smaller at each hop approaching the destination.

A second scenario considers the high likelihood of random forwarding at the beginning of the path, as can be seen in Figure 2c. This could happen because the distance to the destination, which is the denominator of Equation (1), is long at that point. Recall that a large value in the denominator of Equation (1) increases the probability *p* of random forwarding. Figure 2c includes two possible paths originated by random forwarding operations at the first hop. These paths are labeled as “1” with a continuous line and “2” with dotted lines. Simulated annealing can also generate long paths, when in fact there is a shorter path in the topology. In Figure 2c, Node O1 in path “1” could forward randomly to O2 (red arrow labeled as “3”) and follow the second path, which is longer than path “1”. To conclude our example, we point out that the controlled random forwarding of simulated annealing can fail in our example. For instance, simulated annealing could generate the bad paths S→B1→P5 or S→B1→P5→P1. Nevertheless, simulated annealing provides the chance of avoiding the local minimum at G1 that would be unavoidable if the routing protocol made just greedy decisions.

Summarizing, with simulated annealing, if the best candidate changes the distance to the destination little with respect to the distance from the current node or/and the current node is far away from the destination node, then the probability *p* (of choosing a node farther than the current node) will be high. This probability *p* depends on a tuning factor *γ*.

## 4. Geographical Heuristic Routing Protocol

In this section, we present our proposal of the geographical routing protocol named the Geographical Heuristic Routing (GHR) protocol. GHR follows the DTN approach of carrying the packet when there is not a suitable next forwarding node. Moreover, our routing protocol can use any routing criteria to select the next forwarding node to make forwarding decisions at each hop.

The GHR protocol combines Tabu search and the Simulated Annealing (SA) adaptation summarized in the previous Section 3 to avoid loops and select the next node with a certain degree of randomness, respectively. GHR does not need to add any information to the hello messages. Algorithm 2 shows the procedure of our proposed GHR. First, it needs five input parameters:

- The packet *p* to extract its Tabu list *τ*.
- The set of possible destination nodes, Dst, according to the anycast address of the destination.
- The Boolean variable Tabu that indicates if Tabu routing has to be used. This means that a node cannot forward packets to the nodes in the Tabu list *τ*.
- The Boolean variable First to select the first neighbor that meets the routing conditions.
- The forwarding factor *α* affects the probability of selecting a random legal neighbor, which is closer to the destination than the current node *v* to forward the packet. The factor *α* plays the role of *γ* in Equation (1) for the forwarding Simulated Annealing (SA) explained in previous Section 3.2.2. If α=0, then the next forwarding node will be selected randomly among the legal neighbors (i.e., random forwarding). On the contrary, if this factor α→+∞, then the best neighbor closer to the destination than the current node will always be selected (i.e., best forwarding). For 0<α<+∞, we obtain probabilities *p* between zero and one, which provides randomness to the forwarding decision.
- The recovery factor *β* tunes the probability of avoiding the “carry and forwarding” approach in favor of forwarding a packet to a legal neighbor that is farther from the destination than the current node. The factor *β* plays the role of *γ* in the Equation (1). When β=0, the routing protocol will always select a legal neighbor, if there is any, as the next forwarding node. On the other hand, when β→∞, carry and forwarding is always applied. Similar to the forwarding factor, if 0<β<+∞, then the GHR protocol uses the recovery simulated annealing, which forwards the packet to a farther node to the destination with a probability 0<p<1, computed according to Equation (1).

The first operations performed by the GHR are the following:

1. Extraction of the Tabu list *τ* from the packet *P* and
2. Set the initial value of the decision variables $v_{\mathrm{nh}},\;v_{f}$ and $v_{r}$ to the current node ID (Lines 1 and 2 of Algorithm 2).

**Algorithm 2** Our proposed Geographical Heuristic Routing (GHR) protocol.GeographicalHeuristicRouting (P,Dst,Tabu,First,α,β)**Require:** a packet *P*, destination set Dst, use of Tabu, use of First node, forwarding factor *α*, recovery factor *β*, current veh *v***Ensure:** Select the best neighbor *v*_nh_ to reach a member of the destination set *v*_dst_.1:τ← GetTabuList(P,state) {if Tabu=False then τ=∅}2:$v_{\mathrm{nh}}\leftarrow v,\;\;v_{f}\leftarrow v,\;\;v_{r}\leftarrow v$ {Initializing next hop variables}3:$s_{v_{f}}\leftarrow -\infty ,\;\;s_{v_{r}}\leftarrow -\infty$4:d${}_{v-v_{f}}\leftarrow$0, d${}_{v-dst_{f}}\leftarrow$0,  d${}_{v-v_{r}}\leftarrow$0,  d${}_{v-dst_{r}}\leftarrow$0 {Initializing distance variables for SA procedures}  5:θ← U[0, 1]6:L(N(*v*),*v*) ←∅ {Initial empty set of legal neighbors of *v* closer to $v_{\mathrm{dst}}$}7:**for all** $v_{\mathrm{dst}}\in Dst$ **do**8: d${}_{v}\leftarrow$ D($v,v_{\mathrm{dst}}$)9: **for all** n∈ N(*v*) **do**10:  **if** n∉τ **then**11:   **if** IsLegal(n)=True **then**12:    d${}_{v-n}\leftarrow$ d${}_{v}$ - D($n,\;v_{\mathrm{dst}}$)13:    $s_{n}\leftarrow$ ComputeMetric(*n*)14:    **if** d${}_{v-n}>$0 **then** {Forwarding phase}15:     L(N(*v*),*v*) ← L(N(*v*),*v*) +n16:     **if** $s_{n}>s_{v_{f}}$ **then**17:      v${}_{f}\leftarrow n,\;\;s_{v_{f}}\leftarrow s_{n}$,  d${}_{v-v_{f}}\leftarrow$ d${}_{v-n}$  d${}_{v-dst_{f}}\leftarrow$ d${}_{v}$ {Set the current best vehicle as forwarding vehicle}18:      **if** First=True **then**19:       $v_{\mathrm{nh}}\leftarrow v_{f}$20:       **goto End**21:    **else** {Revocery phase: To use a farther vehicle to dest. $v_{dst}$ than *v*: d${}_{v-n}\leq$0}22:     **if** $s_{n}>s_{v_{r}}$ **then**23:      $v_{b}\leftarrow n,\;\;s_{v_{r}}\leftarrow s_{n}$,  d${}_{v-v_{r}}\leftarrow$ d${}_{v-n}$   d${}_{v-dst_{r}}\leftarrow$ d${}_{v}$ {Set the best legal vehicle is not closer to $v_{\mathrm{dst}}$ as recovery vehicle}24: **end for**  25:**end for**  26:**if** L(N(*v*),*v*)≠∅ **then** {Forwarding phase: d${}_{v-n}>$0}27: $p=e^{-\alpha d_{v-v_{f}}\;/\;d_{v-dst_{f}}}$28: **if** p >θ **then**29:  $v_{f}$ := S${}_{\mathrm{Random}}$(L(N(*v*),*v*)) {Set a random legal neighbor as forwarding vehicle}30: $v_{\mathrm{nh}}$ = $v_{f}$31:**else** {Recovery phase: d${}_{v-v_{b}}\leq 0$}32: **if** $v_{r}\neq v$ **then**33:  $p=e^{\beta d_{v-v_{b}}\;/\;d_{v-dst_{r}}}$34:  **if** p >θ **then**35:   $v_{\mathrm{nh}}$ = $v_{r}$36:**End:**37:**if** v${}_{\mathrm{nh}}\neq v\wedge Tabu=True$ **then** {The packet will be forwarded}38: UpdateTabuList (τ,v,P)39:**return** $v_{\mathrm{nh}}$


The Tabu list will be empty if the Tabu option is not enabled, and therefore, there will not be any forbidden neighbor as the next hop. Regarding $v_{\mathrm{nh}}$, it stores the next forwarding node. It can be equal to the selected node $v_{f}$ of the forwarding phase, to the selected backup node $v_{r}$ in the recovery phase or it can store the initial value *v* because no neighbor was chosen to forward the packet *P*.

Secondly, GHR initializes to a very low value the best scores $s_{v_{f}}$ and $s_{v_{r}}$ of the selected node in the forwarding and recovery phases, respectively (line 3). Then, the variables $d_{v-dst_{f}}$, $d_{v-dst_{r}}$, $d_{v-v_{f}}$ and $d_{v-v_{r}}$ are initialized to zero (Line 4 of Algorithm 2). The two former variables, i.e., $d_{v-dst_{f}}$, $d_{v-dst_{r}}$, store the difference of distances to destination from the current node *v* and the best forwarding $v_{f}$ and recovery $v_{r}$ candidate neighbors, respectively. The distance of the current node to the destination member for which the best forwarding and candidate nodes got their score are kept in $d_{v-dst_{f}}$ and $d_{v-dst_{r}}$, respectively. These four variables related to distances or the difference of distance to the destination are used in the computation of a probability *p* of using a random forwarding node or a recovery node, depending on the operation phase of GHR.

After that, a random uniform value between zero and one is stored in the variable *θ* (Line 5). The probability of random or recovery forwarding is compared to the value *θ* to decide if any of these options is performed. Next, in Line 6 of Algorithm 2, the set of legal neighbors L(N(*v*), *v*) is initialized as an empty set. L(N(*v*), *v*) stores the legal neighbors *n*, which are closer to some destination member $v_{\mathrm{dst}}\in Dst$ than the current node *v*.

GHR searches the best forwarding $v_{f}$ and recovery $v_{r}$ nodes considering all of the members of the destination set Dst. The GHR algorithm goes through the list of destination members (see the “for” loop from Lines 7 to 25 of Algorithm 2), seeking the forwarding and recovery nodes with the highest scores among legal neighbors. Hence, GHR makes an exhaustive search and chooses the neighbor with the best metric over all of the other neighbors and members of the destination set.

More specifically, for each destination member $v_{\mathrm{dst}}$, GHR searches among the neighbors of current node N(v) (see the “for” loop from Lines 9 to 24) if any of them improves the current scores $s_{v_{f}}$ and $s_{v_{r}}$ of $v_{f}$ and $v_{r}$, respectively. In the selection process, the first step is to obtain the distance from the current node to the destination member $v_{\mathrm{dst}}$, which will be stored in d${}_{v}$ (Line 8). Then, GHR checks if the neighbor *n* under evaluation is not in the Tabu list *τ* (Line 10) and that it is legal (Line 11). Only if node *n* is not in the Tabu list and it is legal, the algorithm considers neighbor *n* as a possible next forwarding node. Otherwise, the node is discarded by the selection process.

After verifying the eligibility of a neighbor *n*, the GHR protocol calculates the difference of distances, called d${}_{v-n}$, between the current node *v* to the destination member $v_{\mathrm{dst}}$ (i.e., $d_{v}$) and the candidate node *n* to the destination member $v_{\mathrm{dst}}$, i.e., $D(n,v_{\mathrm{dst}})$ (see Line 12). In addition, the algorithm computes the routing metric score of the neighbor *n* (Line 13). If d${}_{v-n}\geq 0$ in Line 14, this means that *n* is closer to $v_{\mathrm{dst}}$ than the current node *v*; therefore, *n* is a candidate to be the next forwarding node $v_{f}$, and the neighbor *n* is added to the set of legal neighbors L(N(*v*), *v*) (Line 15). Otherwise (i.e., d${}_{v-n}<0$ in Line 21), the neighbor *n* is considered as a possible recovery node. We would like to highlight that, if a neighbor *n* is a legal forwarding node for more than one destination member, then that neighbor *n* will be included in the set of legal neighbors as many times as it was considered legal in the selection process. Hence, the probability of that node *n* being chosen in a random selection (Line 29) will be higher than for a node that is legal only for one destination member.

If the neighbor is closer to the destination (d${}_{v-n}\geq 0$), the score of neighbor *n* is compared with the current best forwarding score $s_{v_{f}}$ (Line 16). If it is higher than the current best score, then the neighbor *n* becomes the best forwarding node $v_{f}$ and the best forwarding score $s_{v_{f}}$, and the difference of distance d${}_{v-v_{f}}$ between current node *v* and best candidate $v_{f}$ to the destination is updated to d${}_{v-n}$ and $s_{n}$, respectively (Line 16). When the first option is set to true, the first legal forwarding node $v_{f}$ is selected as the next forwarding node $v_{\mathrm{nh}}$ (Lines 18 and 19), and the searching stops and goes to the final step of the algorithm.

When the neighbor *n* is considered to be in the recovery phase (Line 21 of Algorithm 2), if the neighbor score $s_{n}$ is higher than the current best recovery score $s_{v_{r}}$ (Line 22), then *n* is the new best recovery node $v_{r}$, and the corresponding score and distance variables are updated (Line 23).

After that, the GHR protocol searches for the best forwarding node considering all of the members of the destination set; it checks whether the set L(N(*v*), *v*) is empty or not (Line 26 of Algorithm 2). If it is not empty, which means that there are neighbors closer to the destination, then a probability *p* is obtained as a function of the forwarding factor *α*, the difference of distances d${}_{v-v_{f}}$ and the distance from the current node to destination d${}_{v-dst_{f}}$ following Equation (1). The purpose of these factors was explained in the simulated annealing of Section 3.2. If p>θ, then the next forwarding node is chosen randomly from the set of legal neighbors (Line 29); otherwise, the next forwarding node is the best neighbor previously stored in $v_{f}$.

When there is no legal neighbor closer to the destination (i.e., L(N(*v*),*v*) = ∅), the algorithm checks if there is a legal recovery node $v_{r}$ different from the initial value of it, which was set to node *v* ($v_{r}\neq v$ in Line 32). If there is some recovery node $v_{r}$, then it will be farther from the destination (i.e., $d_{v-v_{b}}\leq 0$). A probability *p*, computed as in the forwarding case (Line 33), decides if the packet *P* is forwarded to $v_{r}$ (p>θ in Line 34) or if on the contrary, the packet is stored in a buffer. Notice that there is no negative sign in the equation of Line 33 because $d_{v-v_{b}}\leq 0$.

It is worth pointing out that we employ the random uniform number *θ* to decide if the probability computed with Equation (1) becomes true in the routing process. Since *θ* changes its value at each forwarding operation, the routing decision depends on the probability of randomness *p* and on the current value of theta. This makes random the decision of selecting a random node or a recovery node, which is the idea of simulated annealing. For instance, in the forwarding phase, even for high values of *p*, there is a chance of not randomly routing a packet because the current value of *θ* is higher than *p*. If theta were a fixed value, then the algorithm would use random routing or a recovery node only depending on the value of *p* computed through Equation (1). This would make decisions not random at all, contrary to the simulated annealing principle.

Finally, if Tabu routing is being used (i.e., Tabu=True) and the packet will be forwarded to some neighbor (i.e., $v_{\mathrm{nh}}\neq v$), then the current node *v* is added to the top of the Tabu list *τ* according to the procedure described in Section 3.2.1.

## 5. Performance Evaluation

In this section, we present the performance evaluation of our routing proposal GHR. First, we study the forwarding options and the recovery setup of GHR in order to find the most suitable configuration for this protocol. After that, we compare the best configurations of GHR using different scoring algorithms against an adaptation for VANETs of the classical topological routing protocol Ad-hoc On demand Distance Vector (AODV) routing protocol [21].

### 5.1. Simulation Settings

To carry out our performance evaluation, we use a simulation scenario for a reporting service, like traffic and/or environmental measurements, in a multi-hop VANET. These kinds of applications are not delay sensitive and can tolerate a moderate percentage of packet losses because reports coming from close enough positions may contain redundant information.

We carried out the simulations using the Estinet Network Simulator [22]. Estinet is a simulator that includes the standard IEEE 802.11p and a simple and accurate way to design VANET realistic scenarios.

We considered three different amounts of vehicles: 100, 150 and 250 vehicles, which correspond to densities of 67, 100 and 166 vehicles per km${}^{2}$, respectively.

For the evaluation of our proposals, we run 20 simulations per each vehicle density using different movement traces to present the figures with confidence intervals of 95%.

**Figure 3:**
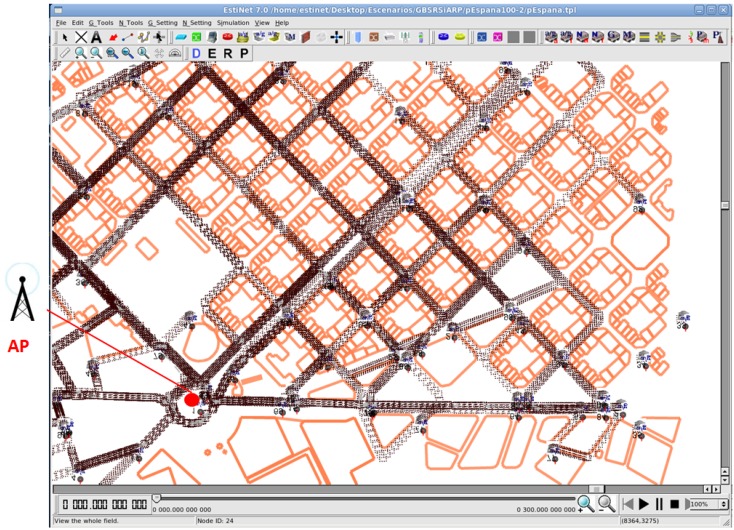
Simulation scenario. Eixample district of Barcelona, Spain, with an Access Point (AP). Buildings (orange lines) from OpenStreetMap are included.

We used a real scenario of 1.5 km${}^{2}$ from the Eixample district of Barcelona (see Figure 3). In our realistic scenario, the mobility model was obtained with CityMob for Roadmaps (C4R) [23], a mobility generator that uses the SUMO engine [24]. C4R is able to import maps directly from the OpenStreetMap [25] and to generate NS-2 mobility traces. C4R considers random origins and destinations for each vehicle’s trip in the simulation area. These points can be located with a higher probability in areas specified by the user. In addition, the path between the start and end points of a vehicle’s trip is computed through Dijkstra’s algorithm in a directed graph formed by the streets and their directions in the map (as a GPS-based navigation system computes a route). We exported the NS-2 traces to be compatible with Estinet and the buildings information using our own translating software, available at [26]. Furthermore, the scenarios have building information (orange lines in Figure 3) extracted from the OpenStreetMap using the SUMO tools.

There was one fixed destination, the Access Point (AP) in Figure 3, that receives the vehicles’ traffic information (e.g., traffic reports, infraction notifications, event of an accident, etc.). We used a single AP in the scenario because in this way, we obtained a long range of route lengths, which depend on the position of the source vehicles in the scenario. All nodes sent 1000-byte packets every *T* seconds to the unique destination during 300 s. *T* follows a uniform distribution from 2 to 6 s. We point out that these two settings (i.e., a single AP and all vehicles generating traffic) are adverse for successful multi-hop transmissions because they make medium access contention very challenging, and therefore, collisions and the associated packet losses are more likely to happen. Moreover, packet transmissions from long paths due to the single AP in the scenario increase the chances of packet losses. For these reason, we consider the results presented in our paper a “worst case scenario” in the evaluation of the performance of our routing proposal.

Simulations were carried out using the IEEE 802.11p standard on physical and MAC layers. Moreover, we performed the simulations using the adaptation of the Contention Window (CW) mechanism proposed in [27] to adapt the CW in a smoother way especially designed for VANETs for congestion control. We incorporated the Coherent Automatic Address Resolution (CAAR) explained in [28]. CAAR adds the MAC address of a node into its hello messages of the routing protocol. In this way, CAAR avoids the address resolution handshake between nodes of paths to the destination because the couple MAC/IP addresses are received at the same time. Additionally, we enabled a packet filtering based on packet ID in the ad hoc routing protocols proposed in [29]. This filter works in a similar manner as the duplicate frame filter of the MAC layer. The routing filter mitigates the propagation of unintentional copies generated by the local recovery procedures of routing protocols when a frame transmission fails because of the loss of an ACK frame.

All of the figures are presented with Confidence Intervals (CI) of 95%, obtained from 20 simulations per each density value, a GHR setup combination and using different movement traces per each simulation. Table 1 summarizes the main simulation settings.

In the first step, we employed MMMR [20] in the core of our routing proposal GHR to evaluate the different configurations that GHR can provide. Multi-Metric Map-aware Routing (MMMR) is a position-based, traffic-aware and delay-tolerant protocol based on the Greedy Perimeter Stateless Routing (GPSR) [8]. MMMR considers four metrics instead of only one as GPSR to select the next forwarding hop among its neighbors. These four metrics evaluate the distance to the destination, the vehicle trajectory, the vehicle density and the available bandwidth. MMMR will be in charge of scoring the neighbors and choosing the best one among them.

As the second step, we used the best configurations of GHR, found with MMMR as the score algorithm, to test these configurations with a classical DTN routing protocol, named Greedy-DTN in [4], in the scoring procedure of GHR. The greedy-DTN protocol is a variation of GPSR that replaces the perimeter mode by the carry and forwarding approach. The greedy-DTN protocol uses only distance to destination to select the next forwarding node. We present this comparison to provide an idea of the role of the scoring algorithm. Even more, this shows how GHR can be easily used for any DTN position-based routing protocol that makes routing decisions in a hop by hop fashion. In addition, we have compared these two protocols, i.e., GHR-MMMR, GHR-greedy-DTN, against a modified version of AODV for VANETs called irresponsible AODV [33], which was especially adapted for VANETs because it reduces the number of route request signaling messages to establish an end-to-end path.

Notice that the Euclidean distances used by the three protocols, i.e., MMMR, greedy-DTN and iAODV , are computed through the positions provided by the GPS on-board units of the vehicles. Every node includes its own position in the hello messages received by its neighbors. It is known that the GPS positions have an error, whose radius typically ranges from zero to 10 meters [34]. In our simulations, every time a node queries its position from the GPS unit, the simulator adds a uniform random error with a radius from zero to 10 meters to the exact position. This way, we mimic what happens in realistic GPS devices.

### 5.2. Statistical Procedure

The performance analysis of our heuristic protocol GHR is based on four different metrics. These metrics are:

- Packet Delivery Ratio (*PDR*): This is the total percentage of packets (sent from the vehicles) that reach the AP. PDR does not increase due to the reception of copies of a packet. Hence, this metric measures the effectiveness of the routing protocol in terms of different delivered packets.
- Average end-to-end delay: This is the average time elapsed from the transmission of a packet until it arrives at the destination (computed either for the original packet or for a copy).
- Average number of hops: This is the average number of hops that a packet needs to reach the AP. This average includes the hops performed by the original packet and by its copies.
- Percentage of idle time: This is the average of the idle time sensed by a node measured in 1 s. A node senses the channel as idle when it is not transmitting nor receiving a packet and given that the interference level is below the antenna sensitivity. Notice that a higher idle time sensed by the nodes leads to more bandwidth available in the channel to transmit more information. An estimation of the available bandwidth derived from the idle time measure could be done using the models proposed by [35] or [36].

We are interested in knowing if the differences in the performance metrics listed above are statistically significant or not. Furthermore, we want to determine if the presence of differences depends on the vehicle density and/or on other factor, for instance the use of the Tabu list in routing operations. To do this, we carry out MANOVA [37] (Multivariate Analysis of Variance ) tests over the data using the statistical software SPSS [38]. We use MANOVA to consider the inherent correlation among the performance metrics when they are not independent from each other.

For the MANOVA tests, we report the value of the statistics Wilk’s Λ and F, which allow us to obtain a probability called the *p*-value. A *p*-value is compared with a threshold named the significance level to determine if the simulation findings are statistically relevant (i.e., the *p*-value is lower than the significance level). We use for our test a *p*-value = 0.05 for the significance level. We divided the statistical procedure analysis into three steps:

Step 1 Tests to determinate interactions among the identified factors for each analysis over the four performance metrics. If an interaction is detected, then the performance differences in a factor will depend on the combination of the factors involved.
Step 2 Tests to determine if there is statistical difference in metrics for each one of the groups in which the dataset was divided because of the presence of interactions. If there is not a statistical difference in a metric, then this means that this metric behaves similarly under the different levels of the studied factor (i.e., heuristic technique). Otherwise, the test tells us that there is a difference, but it does not indicate between which levels of a factor this difference is present.
Step 3 Pairwise comparisons for a metric. If the previous tests determine a significant difference in a metric with a certain factor, then we run a *t*-test pairwise comparison among the different levels of the factor under analysis. The objective of this step is to establish the performance relation between levels.

By following the above test order, we are able to provide a detailed and accurate analysis of the advantages and costs of our proposals. Next, we present our analyses and statistical results for each routing protocol separately.

### 5.3. Evaluation of the Forwarding Phase in GHR

To evaluate the forwarding phase of our Geographical Heuristic Routing (GHR) protocol, we distinguish three factors that could affect the performance of the GHR. These factors are:

1. The vehicle density of the scenario (VD).
2. The use of a Tabu list in the routing (*T*).
3. The forwarding technique (FT).

We compare the four different ways that GHR uses to select the next forwarding node: the best legal neighbor (α→∞), a random legal neighbor (α=0), randomly according to Simulated Annealing (SA) (α=3) or the use of the first legal neighbor. Following the three-step procedure described in previous Section 5.2, we have to analyze the results for each density separately because there is a significant three-way interaction FT×T×VD (Step 1) with a *p*-value = 0.001 (Wilk’s Λ = 0.377 and F (24,92) = 2.41).

Table 2 shows the results of the further interaction test between the use of Tabu *T* and FT for the GHR protocol (Step 1). First, we perform the so-called “all together” test in which we evaluate the interaction between the two factors considering the correlation among the six performance metrics. If the *p*-value < 0.05 for this test, we further carry separate interaction tests for each metric. In Table 2, we can see that for the medium and the high density scenarios, the “all together” test has a *p*-value under the threshold, so independent interaction tests per metric need to be performed in those cases. The results of the interaction tests for each metric indicate that the seeking of performance differences in the forwarding technique has to differentiate if tabu were used or not in the routing in the medium and high density scenarios (150 and 250 vehicles, respectively) to compare the average number of hops. There is also this significant interaction (i.e., FT×T) for the evaluation of the percentage of idle time in the scenario of 250 nodes.

The test results to determine if there are differences in the performance metrics, according to Step 2 of the statistical procedure of Section 5.2, are shown in Table 3. For this analysis, the data of the metrics were grouped according to the results of the interaction tests analyzed previously. This means that in most of the cases, we performed only one test per metric in each density without differentiating whether Tabu is enabled during the simulation. We labeled these cases as “together” in the Tabu column. All of the *p*-values in this table are lower than 0.05, excluding the percentage of packet losses in the medium density scenario (150 vehicles) and the average end-to-end delay for the three vehicle densities. Hence, the use of different forwarding techniques (i.e., α→∞, α=0, α=3 or First) produces a statistically-significant change among them in all performance metrics, and therefore, they require pairwise comparisons to analyze the differences. These pairwise comparisons are not needed for the average delay metric in all densities and for the percentage of packet losses in the medium density scenario, because there are no differences among the forwarding techniques in these cases.

Following Step 3 of the statistical analysis, Table 4 shows the results of the pairwise comparisons among the forwarding techniques (i.e., (α→∞, α=3), (α→∞, α=0), (α→∞, First), (α=3, α=0), (α=3, First) and (α=0, First)) in which there is no statistical significance for a particular metric (i.e., *p*-value ≥ 0.05). In these cases, the average values of the metrics are very similar and can be considered statistically the same.

The rest of the results of the pairwise comparison tests (not included in Table 4), e.g., the percentage of packet losses for the high density scenario when Tabu is not enabled, obtained *p-*values < 0.05. Those *p*-values indicate that the forwarding technique (i.e., best node, SA, random or first) has an impact on the values of the metrics.

**Figure 4:**
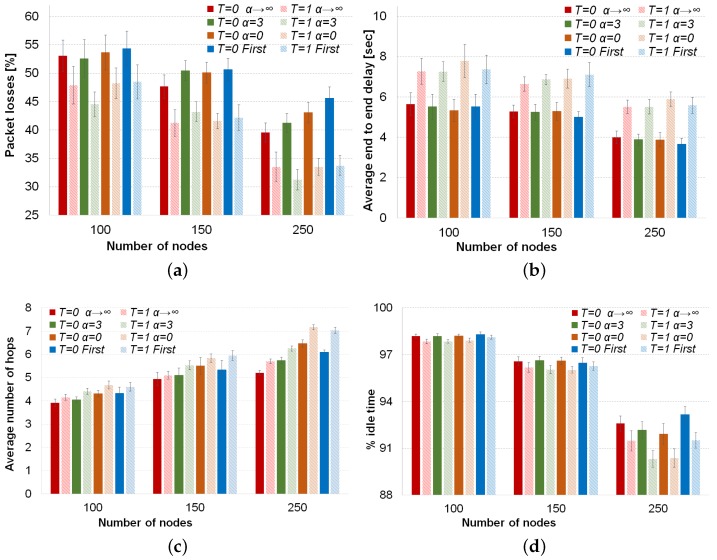
Performance evaluation of the forwarding techniques (FT) included in our proposed GHR. T=1 means that our Tabu routing is used. The four FT are: best node selection (α→∞), simulated annealing (α=3), random forwarding (α=0) and first legal node (First). (**a**) Percentage of packet losses; (**b**) average end-to-end packet delay; (**c**) average number of hops; (**d**) percentage of idle time.

The comparisons of average values of the four performance metrics with the different forwarding techniques are depicted in Figure 4. Considering the previous statistical analysis, we continue analyzing the behavior of forwarding techniques in the metrics.

Firstly, the use of our Tabu list, which consists of the three last nodes in the packet path, decreases the percentage of packet losses considerably. This descent in the packet losses goes from 6% in low density scenario till around 10% with high vehicle density, as can be seen in Figure 4a. The reason for this improvement is that our Tabu list provides memory to the routing decision. This memory helps to avoid neighbors of the current node already visited by the packet that otherwise could be selected again to forward the packet. Therefore, the Tabu list avoids loops and helps to consider other possible next forwarding nodes. On the other hand, the use of our Tabu routing proposal increases the average number of hops (see Figure 4c) by around 0.6 hops on average. Forbidding nodes as next hops forces nodes to search other path that might be longer. Moreover, the average end-to-end delay increases around 2 s for the low vehicle density scenario and 1.5 s for the high density scenario. The higher delay is because of the longer paths. In addition, since the amount of possible next forwarding nodes decreases due to the list of prohibited nodes (i.e., Tabu list), the carry and forwarding procedure is used more often, increasing the average delay of packets. Finally, the % of idle time sensed by a node decreases when our Tabu routing is enabled. In Figure 4d, it can be seen that as the vehicle density increases, the difference between using or not using Tabu in the percentage of idle time increases, as well, reaching 2% in the scenario with 250 vehicles. There are two reasons for the decrease of the percentage of idle time; first, the higher number of hops needed by Tabu to reach the destination; second, it is important to notice that the use of a Tabu list is overhead to be carried by each packet until reaching the destination. This means more bandwidth utilization for a packet transmission at each hop in longer paths than when Tabu is disabled.

Nevertheless, the slightly higher delay and lower idle time are not so important compared to the noticeable lower losses achieved when the Tabu list is used. Thus, this trade-off clearly shows benefits in favor of the Tabu list.

Regarding the behavior of the forwarding techniques, as can be seen from Figure 4a, in low vehicle density, the forwarding inspired in simulated annealing (α=3) has a slightly and statistically significant improvement (around 2%), compared to the other three approaches. Indeed, these three forwarding approaches, i.e., best node, random and first legal, behave similarly according to our statistical analysis (see *p*-values > 0.05 for packet losses and 100 vehicles in Table 4). For the medium density, there is not a statistically-significant difference among the forwarding techniques, neither when the Tabu is used, nor when this option is disabled (see second row in Table 3). In the high density scenario, the behavior is different and depends on if Tabu routing is being used or not. When Tabu is not used, it is clear from Figure 4a that the selection of the best neighbor gets the lowest percentage of packet losses. On the other hand, a complete random selection or the selection of the first legal neighbor as the next forwarding node has the worst % of packet losses. The reason lies in the high number of hops that these two forwarding techniques use to reach the destination. When Tabu routing is used in the high density scenario, the behavior of the routing techniques changes completely. The degree of randomness given by the SA forwarding (T=1, α=3 in Figure 4a) gets the best results. The advantage of this approach is based on not always selecting the best node, which could avoid collisions or link saturation, and on avoiding already visited nodes by using the Tabu list. The other three approaches (i.e., α→∞, α=0 and First) have statistically the same results thanks to the use of Tabu.

As can be seen from Figure 4b, the delay among the four different techniques is the same regardless of the use of Tabu routing for the three vehicle densities. This was confirmed by our statistical analysis, whose results (*p*-values ≥ 0.05) are shown in the average delay section of Table 3. From Figure 4c, we can realize that applying a forwarding factor α<∞, the average number of hops increases as is expected because the randomness in the forwarding increases, as well. Therefore, the highest number of hops is always obtained by a complete random selection (α=0), and the lowest hop count takes place for the best selection (α=∞). When Tabu is used or in a low vehicle density, the selection of the first legal node needs as many hops as a random selection (see the *p*-value ≥0.05 of the pairs (α=0, First) in the average number of hops in Table 3).

The percentage of idle time depicted in Figure 4d depends on the forwarding mechanism. The selection of the first legal neighbor has a high percentage of idle time for the three densities. This reveals a better use of the available bandwidth of this forwarding mechanism in spite of the high number of hops that the First strategy needs to operate. The reason is that the First strategy prefers to use recent updated neighbors, which have the most stable links, and therefore, will have a higher number of successful transmissions at the first attempt than other approaches, like best node or random selection. In fact, this technique reaches the highest value for the medium density scenario (150 nodes), while the other techniques obtain *p*-values ≥ 0.05 in the pairwise comparisons of idle time; see Table 4. In the high density scenario, the First strategy has the same high level of idle time as the best node selection (α→∞), while random (α=0) and SA (α=3) have the lowest level of idle time because they perform more hops than the classical selection of the best node. However, it is worth noting that simulated annealing forwarding obtains the lowest percentage of packet losses with Tabu for this high vehicle density.

### 5.4. Evaluation of the Recovery Phase in GHR

In this section, we evaluate the performance of the recovery factor *β*. This factor allows the routing protocol to forward a packet to a node farther from the destination than the current node instead of keeping the packet in the buffer until a better next forwarding node appears. For this evaluation, we consider only the forwarding techniques best neighbor (α→∞) and the simulated annealing forwarding (α=3), both with Tabu routing enabled. The reason for using only these two strategies in the tests of the recovery phase is that SA forwarding has the lowest percentage of packet losses in the three densities of vehicles, and the selection based on the best neighbor is the classical approach in geographical routing. Moreover, the other two techniques, i.e., random forwarding (α=0) and first legal neighbor, behave similarly to the best node selection. They do not outperform the criterion of the best node in terms of packet losses and delay.

For this part of our study, we work with three factors that could affect the performance of the recovery phase of GHR. These factors are:

1. The vehicle density of the scenario (VD).
2. The forwarding technique (FT).
3. The recovery technique (RT), which depends on the *β* factor. We consider three different values for *β*. They are: β→∞, which is the default carry and forwarding approach; β=3 to use a recovery SA; and β=0, which always selects a next forwarding node if the current node has some legal neighbor.

**Figure 5:**
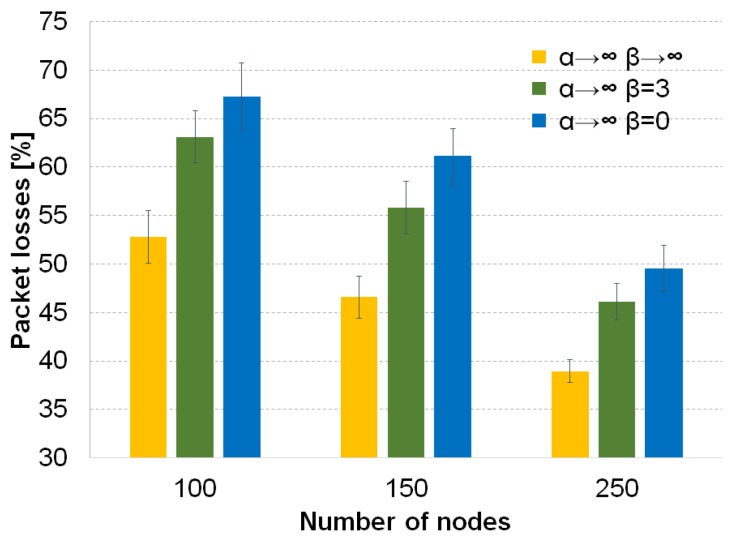
Percentage of packet losses for different values of the recovery factor *β*, using the best node criterion at the forwarding phase without Tabu.

We do not evaluate the use of the recovery factor *β* for the forwarding techniques without Tabu because the packet losses increase significantly for those cases, as can be seen in Figure 5 for the forwarding technique based on the selection of the best neighbor (α→∞). The values of β=3 or β=0 perform poorly because the recovery mechanism creates loops without Tabu. The recovery mechanism selects in most of the cases the previous neighbor that forwarded the packet to the current node. This creates loops, and nodes cannot avoid those nodes because they do not have a track of the previous path followed by the packet.

We have to analyze the results for each density separately because there is a significant three-way interaction RT×FT×VD (Step 1) with a *p*-value = 0.0001 (Wilk’s Λ = 3.41 and F (16,100) = 4.47).

Table 5 shows the results of the further interaction test between the FT and RT for GHR protocol (Step 1). We can see that for the medium and the high density scenarios, the “all together” tests have a *p*-value under the threshold, so an independent interaction test per metric needs to be performed in those cases. The *p*-value < 0.05 in the interaction tests for each metric indicates that the seeking of performance differences in the recovery technique should be done for each forwarding technique (i.e., best neighbor and SA forwarding) separately.

The test results to determine if there are differences in the performance metrics, according to Step 2 of the statistical procedure, are shown in Table 6. All of the *p*-values in this table are lower than 0.05, excluding the percentage of packet losses in the high density scenario (250 vehicles) for the forwarding factor α→∞ (select the best neighbor). Hence, the use of different values for the recovery factor *β* produces a statistical significant change among them in all performance metrics, and therefore, they require pairwise comparisons to analyze the differences.

Table 7 shows the results of the pairwise comparisons among the recovery techniques (i.e., (β→∞, β=3), (β→∞, β=0), (β=3, β=0)) following Step 3 of the analysis, in which differences between *β* values are not statistical significant for a particular metric (i.e., *p*-value ≥ 0.05). In these cases, the average values of the metrics are very similar and can be considered statistically the same.

The rest of the results of the pairwise comparison tests, e.g., the average end-to-end delay in the three vehicle densities, obtained *p-*values <0.05, and we do not include them in Table 4. Those *p*-values indicate that the value of the recovery factor *β* has an impact on the values of the metrics.

**Figure 6:**
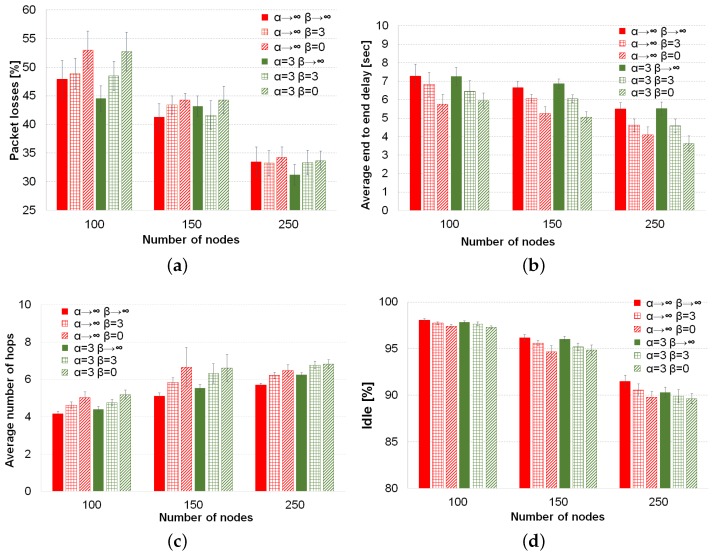
Performance evaluation of the recovery techniques (RT) included in our proposed GHR. The three RT are: carry and forwarding (β→∞), Simulated Annealing (SA) (β=3) and aggressive recovery (β=0). We compare them with two forwarding techniques (FT): best node selection (α→∞) and SA (α=3). (**a**) Percentage of packet losses; (**b**) average end-to-end packet delay; (**c**) average number of hops; (**d**) percentage of idle time.

The comparisons of average values of the four performance metrics with the different forwarding techniques are depicted in Figure 6.

The behavior of the recovery techniques in terms of packet losses can be seen in Figure 6a. The carry and forwarding strategy (β→∞) is the best option for the low vehicle density. As the value of *β* increases, the percentage of packet losses becomes significantly higher in this density. In the intermediate density, for the classical forwarding to the best node (α→∞), SA recovery and the aggressive recovery (β=0) reach the same level of packet losses (see the first row in Table 7) between them, but they do not improve the default carry and forwarding (β→∞). Only the SA recovery (β=3) has a better performance than the default carry and forwarding for SA annealing forwarding (α=3) in the intermediate density. Moreover, in this scenario (150 vehicles), this approach (α=3, β=3) has the same packet losses as the default selection of the best node with carry and forwarding (α→∞, β→∞). In the high vehicle density, the three recovery mechanisms behave very similarly as the classical forwarding (α→∞). On the other hand, for the SA forwarding process (α=3), carry and forwarding (β→∞) is again the best strategy when SA is used. However, contrary to the low density scenario, the other two recovery values of *β*, which are similar between them (see the third row in Table 7), are close to the percentage of packet losses of SA forwarding with β→∞.

The average end-to-end delay is related with the value of the recovery factor *β*. As can be seen from Figure 6b, when the *β* value decreases, the average end-to-end delay decreases, as well, for the two forwarding techniques. The reason is that the low *β* values produced use the buffer less often than carry and forwarding, which is the main cause of high delays. β=0 has the lowest delay because it uses the buffer only when there is not any legal neighbor. This aggressive recovery leads to a decrease around two seconds with respect to the carry and forwarding technique in the three vehicle densities. More importantly, this decrement comes with none or very little degradation in the percentage of packet losses for intermediate and high densities, respectively.

From Figure 6c, we can realize that applying a recovery factor β<∞, the average number of hops increases because the packets are forwarded more times than with carry and forwarding. When *β* decreases, the increment in the average of hops is statistically significant (see the *p*-values <0.05 in the average number of hops section in Table 6). In fact, the are only two *β* values that reach the same number of hops, the SA recovery (β=3) and the aggressive recovery (β=0) for classical forwarding (α→∞) in the intermediate density. Notice that the differences in the average number of hops between the classical approach (β→∞) and the other *β* values examined in this section is up to 1.5 hops at maximum.

Regarding the percentage of idle time depicted in Figure 6d, it follows exactly the same behavior described for the average number of hops. That is, while the probability to forward a packet instead of keeping it in the buffer (low *β* values) increases, the percentage of idle time decreases. Only in the high density scenario and for SA forwarding (α=3), the idle times sensed by SA recovery (β=3) and aggressive recovery (β=0) are the same. Nevertheless, the maximum difference between the conservative carry and forwarding and the other mechanisms is at maximum 2% for the three vehicle densities.

To summarize the performance evaluation of our Geographical Heuristic Routing (GHR) protocol, we have found that SA forwarding with carry and forwarding (α=3, β→∞) is the best option for low density areas, because it shows the best percentage of packet losses in this vehicle density. In the intermediate density, SA forwarding and recovery (α=3, β=3) obtains the same level of packet losses as traditional forwarding (α=→∞, β→∞) with a lower delay. For this reason, we choose this configuration for medium vehicle density. Finally, we consider that SA forwarding with aggressive recovery (α=3, β=0) is appropriate for high vehicle density scenarios. This GHR setup gets the lowest average delay and only decrements around 2% the best packet delivery ratio in this density, achieved by SA forwarding (α=3, β→∞). We will use these specific configurations, which depend on the vehicle density, to compare GHR with MMMR, greedy-DTN and iAODV in the next section.

### 5.5. Performance Comparison

In this section, we show how an adequate configuration of GHR can enhance the performance of geographical routing protocols. In addition to using the MMMR routing procedure to score the neighbors of a node, a case widely studied in the two previous sections, in this section, we employ a basic routing criterion, which is based only on the distance to the destination to choose the next forwarding node. This approach is generally called greedy-DTN [4], and it is widely used as a reference for comparison with more sophisticated DTN protocols [4].

Moreover, we compare our proposed GHR with these two routing algorithms, i.e., GHR-MMMR, GHR-greedy-DTN, against a modified version of AODV called irresponsible AODV (iAODV) [33], which was especially adapted for VANETs because it reduces the number of route request signaling messages to establish an end-to-end path. iAODV is a representative example of topology-based routing protocols.

**Figure 7:**
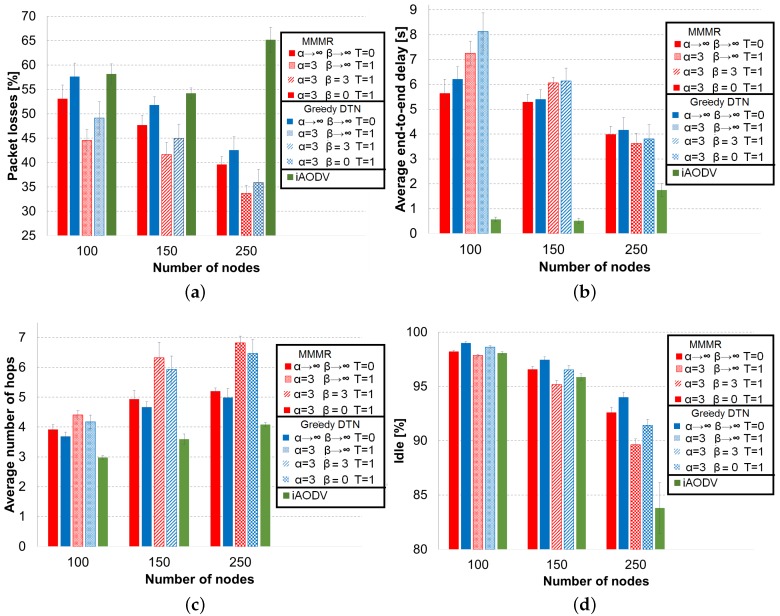
Performance comparison of the best GHR configurations using MMMR and greedy-DTN as the scoring algorithms and iAODV. (**a**) Percentage of packet losses; (**b**) average end-to-end packet delay; (**c**) average number of hops; (**d**) percentage of idle time.

Figure 7 shows the results of the four metrics used in this paper for the three protocols that we have compared, GHR-MMMR, GHR-greedy-DTN and iAODV, with red, blue and green bars, respectively. The default operations of MMMR, greedy-DTN and iAODV are the bars with solid fill (first, second and fifth bars). The results for MMMR and greedy-DTN when they are assisted with the best configuration of GHR, which depends on the vehicle density, are represented in red and blue bars with different filling patterns for each vehicle density.

The percentage of packet losses in Figure 7a lets us see that the default MMMR operation (solid red bar), without GHR assistance, outperforms the default operation of greedy-DTN and iAODV in the three vehicle densities. The reason is the more elaborate scoring metric that considers, in addition to the distance, the trajectory of the neighbor, the vehicle density and the available bandwidth. If the trajectory of a neighbor approaches the destination, then it would be useful if the node implements “carry and forwarding”. A high vehicle density increases the probability of finding a suitable next forwarding hop. Finally, it is important that a neighbor has enough available bandwidth for a packet transmission. However, the reader can notice that the importance of the three additional parameters of MMMR with respect to greedy-DTN decreases when the vehicle density increases because it is easier for greedy-DTN to find good forwarding candidates. Regarding iAODV, we can see that it only reaches the greedy-DTN marks with low vehicle density. In this vehicle density, nodes can build and maintain end-to-end routes without mutual interference due to the low signaling traffic. For the intermediate vehicle density, iAODV is able to decrease the percentage of packet losses with respect to its low density results because it can find more routes and maintain a reasonable signaling overhead since it does not use the aggressive flooding of the route request message of the original AODV. However, iAODV is not able to follow the results obtained by default greedy-DTN nor MMMR, because these two protocols use a simple and effective routing strategy. More importantly, greedy-DTN and MMMR need very few signaling messages to operate compared to iAODV, which turns into idler communication channels for data transmissions. Despite the more efficient route construction mechanism of iAODV, broadcast storms of route request/reply messages arise when the number of vehicles is high. As a consequence, the performance of iAODV decreases considerable contrary to the behavior of the geographical routing protocols, which decreases the percentage of packet losses.

When greedy-DTN and MMMR work with GHR, with tailored configurations according to the vehicle density, both protocols decrease their percentage of packet losses. Nonetheless, the behavior between greedy-DTN and MMMR remains the same. That is, GHR-MMMR outperforms GHR-greedy-DTN in the three vehicle densities, and the difference in the packet losses decrease as the vehicle density increases. It is important to notice that GHR-greedy-DTN improves the default operation of MMMR, i.e., it always selects the best neighbor (α→∞) and it always applies “carry and forwarding” when there is not a closer node to the destination (β→∞). This means that the use of Tabu and simulated annealing is able to improve the straightforward routing criterion of greedy-DTN to improve the MMMR’s results reached by using a combination of four parameters instead of only the distance to the destination.

Figure 7b shows the average end-to-end delay for the received packets. iAODV is the routing protocol with the lowest delay because it establishes end-to-end routes to reach destination. MMMR is able to reduce the delay compared to greedy-DTN because MMMR takes into account the congestion in the path through the available bandwidth metric, the vehicle density to avoid void areas and the trajectory to select nodes that approach the destination. The use of GHR with the routing protocols increases notably the delay with low vehicle density because Tabu is used with carry and forwarding (β→∞). As we explained in previous sections, when Tabu forbids nodes, the set of possible next forwarding nodes is reduced. Consequently, nodes have to apply “carry and forwarding” more often. The delay of GHR configurations in intermediate vehicle density is closer to the default operations of MMMR and greedy-DTN than in low density, because the recovery simulated annealing (β=3) is applied by GHR in intermediate density; hence, “recovery and forwarding” (which introduces high delay) is used less often. Finally, the GHR configuration for high vehicle density decreases the delay compared with the default protocol operation, because this configuration does not apply “carry and forwarding” (β=0).

The average number of hops is depicted in Figure 7c. iAODV needs the smallest number of hops to reach the destination because its algorithm builds a shortest path between the source and destination. On the other hand, MMMR needs more hops than greedy-DTN since the latter only selects the closest node to destination while MMMR considers other parameters and prefers more stable nodes, but requiring more hops to reach the destination. Regarding the GHR configuration, there are two reasons for the increment in the average number of hops. First the use of Tabu forces routing protocols to search alternative routes, typically longer than the ones obtained by the default operations of greedy-DTN and MMMR. The second reason for the increment in the number of hops for GHR configurations in intermediate and high vehicle densities is the use of recovery simulated annealing and aggressive recovery, respectively.

The difference in the percentage of idle time, shown in Figure 7d, is related to the number of hops and the signaling traffic. AODV (green bar) is the most demanding protocol in terms of bandwidth compared to the default operation of greedy-DTN and MMMR (blue and red bars, respectively) because of the high number of signaling messages that AODV needs to operate. This fact is evident in the scenario with high vehicle density in which there is a broadcast storm of signaling messages. Greedy-DTN requires less bandwidth than MMMR because MMMR needs more hops than greedy-DTN. Furthermore, the signaling messages of greedy-DTN are shorter than the ones of MMMR because MMMR needs to transmit information about speed, direction, vehicle density and idle time into the hello messages. Regarding GHR configurations, they have less idle time than default protocol configurations because GHR employs a higher number of hops to reach the destination, and a Tabu list is added to each data packet.

In this section, we have compared the performance of two geographical routing protocols, i.e., MMMR and greedy-DTN, with and without the assistance of the heuristic implemented by our proposal Geographical Heuristic Routing (GHR). The results show that GHR contributes to the improvement in the performance of the geographical protocols. Furthermore, we have shown that geographical routing proposals can reach better results than classical topological routing protocols like iAODV with a reasonable bandwidth consumption.

## 6. Conclusions and Future Work

In this paper, we have proposed a generic Geographical Heuristic Routing (GHR) protocol. GHR is inspired by simulated annealing and Tabu search meta-heuristics [39]. We divide the GHR operation according to the operation in forwarding and recovery. The forwarding heuristics are used when an improvement to reach the destination is feasible. The recovery heuristics are thought to be used with the carry and forwarding approach. GHR can adapt its forwarding criterion depending on the application requirements. We use the traffic-aware MMMR [20] protocol in the core of our proposed GHR to validate and score the neighbors of the current node in the forwarding process. Nonetheless, GHR could use any other routing protocol for the validation and scoring tasks.

An extensive performance evaluation of our proposed GHR indicates that the use of a Tabu list contributes to improving the packet delivery ratio by around 5% to 10%. However, this better performance comes at the price of an additional delay (2 s) because of the more restrictive selection process of the next forwarding node. Nonetheless, the non-real-time applications, which are objective of our work (e.g., report of traffic notifications), could cope with this delay. On the other hand, we show that the classical selection of the best node to forward a packet (i.e., the node with the best metric) and the carry and forwarding recovery are only adequate when the use of Tabu is not possible. On the contrary, if the routing process enables a Tabu list, then a forwarding strategy selection based on simulated annealing and a recovery procedure that does not use the buffer frequently are preferred.

There are some interesting variants of the heuristics presented in this work that we believe are worth testing. Among these variants we highlight the possibility to redefine the numerator of the simulated annealing probability function by considering some function of the distances among candidate nodes. In this way, when candidates are close to each other, random selection would be preferred. Another variant in simulated annealing is not to select, with probability *p*, a random node to forward the packet. Instead, a node will prefer, with probability *p*, a backup node (chosen by some function) rather than the best candidate in the forwarding process.

In our performance evaluation, we have used a single AP, which makes multi-hop communications more challenging. We could achieve a more efficient reporting service through anycast communications. Some of them constitute part of our future work, including: an optimal placement of access points, an efficient procedure to select the member of the anycast group to which the packets will be forwarded and the use of summarizing messages to reduce the rate of reports. Currently, we are developing some algorithms to select the anycast member in the routing protocol based on some of the ideas presented in this work.

## Figures and Tables

**Table 1:** Simulation settings.

Parameter	Value
Map zone	Eixample district of Barcelona
Area	1.5 km × 1 km
Number of nodes	100, 150, 250 vehicles
Vehicle density	67, 100, 166 $\frac{\mathrm{veh}}{{\mathrm{km}}^{2}}$
Mobility generator	SUMO [24]/C4R [23]
Mobility model	Krauss [30]
Max speed	60 km/h
GPS precision radius error	U(0,10) m
Simulation time	300 s
Inter-packet generation time	*T*∼U(2,6) s E(*T*) = 4 s
Packet size	1000 bytes
Path loss model	Empirical IEEE 802.11p radio shadowing [31]
Fading model	Rician (LOS), Rayleigh (NLOS)
Power transmission	23 dbm
Receiving sensing	−82 dbm (∼400 m in LOS)
Routing metric algorithms	MMMR [20] Greedy-DTN [4,32]
Address resolution	CAAR [28]
MAC specification	IEEE 802.11p
Bandwidth	6 Mbps
CW mechanism	CW for congestion control [27]

**Table 2:** MANOVA [37] results for the interaction test between the forwarding technique (FT) and the use of Tabu (*T*) for the forwarding phase of GHR. * The degrees of freedom in the F statistic are 12 and 8. If there is a significant interaction (*p*-value < 0.05) in the “all metrics together” test, then interaction tests per metric need to be performed.

Number of Vehicles	Performance Metric	Wilk’s Λ	F (3,17)	*p*-Value
100	All metrics together *	0.284	1.684	0.234
	All metrics together *	0.103	5.834	0.009
	% of packet losses	0.858	0.941	0.442
150	Average delay	0.661	2.910	0.065
	**Average No. of hops**	**0.335**	**11.272**	**0.0001**
	% of idle time	0.666	2.840	0.069
	All metrics together *	0.113	5.235	0.013
	**% of packet losses**	**0.502**	**0.284**	**0.007**
250	Average delay	0.665	2.858	0.068
	**Average No. of hops**	**0.369**	**9.691**	**0.001**
	**% of idle time**	**0.549**	**4.653**	**0.015**

**Table 3:** MANOVA [37] results of the testing difference in performance metrics among routing forwarding techniques (FT). There is a significant difference when the *p*-value < 0.05. “Together” means that to apply the test, it is not needed to differentiate the use of Tabu in the forwarding techniques.

Metric	Number of Density	Tabu	Wilk’s Λ	F (3,17)	*p*-Value
% of Packet Losses	100	together	0.516	5.306	0.009
	**150**	**together**	**0.666**	**2.846**	**0.068**
	250	No	0.237	18.222	0.0005
	250	Yes	0.456	6.747	0.003
Average delay	**100**	**together**	**0.894**	**0.671**	**0.582**
	**150**	**together**	**0.975**	**0.145**	**0.932**
	**250**	**together**	**0.662**	**2.890**	**0.066**
Average number of hops	100	together	0.163	29.14	0.0005
	150	No	0.198	22.901	0.0005
	150	Yes	0.081	63.96	0.0005
	250	No	0.046	118.0	0.0005
	250	Yes	0.022	253.14	0.0005
% of idle time	100	together	0.332	69.079	0.0005
	150	together	0.203	22.22	0.0005
	250	No	0.137	35.77	0.0005
	250	Yes	0.155	30.979	0.0005

**Table 4:** Pairwise comparison of the performance metrics in which there is a difference among forwarding techniques for the GHR protocol (i.e., *p*-value < 0.05 in Table 3). The table only shows the results for metrics and pairs of forwarding techniques with the absence of statistically-significant differences (i.e., *p*-value ≥ 0.05). “Together” means that it is not needed to differentiate whether Tabu was enabled or not to apply the test.

Metric	Number of Density	Tabu	Pairwise	*p*-Value
% of packet losses	100	together	(α→∞, α=0)	0.317
	100	together	(α→∞, First)	0.276
	100	together	(α=0, First)	0.579
	250	Yes	(α→∞, α=0)	0.98
	250	Yes	(α→∞, First)	0.855
	250	Yes	(α=0, First)	0.741
Average number of hops	100	together	(α=0, First)	0.584
	150	Yes	(α=0, First)	0.142
	250	Yes	(α=0, First)	0.05
% of idle time	100	together	(α→∞, α=0)	0.121
	100	together	(α→∞, First)	0.09
	100	together	(α=3, α=0)	0.067
	150	together	(α→∞, α=3)	0.584
	150	together	(α→∞, α=0)	0.444
	150	together	(α=3, α=0)	0.820
	250	No	(α=3, α=0)	0.096
	250	Yes	(α→∞, First)	0.096
	250	Yes	(α=3, α=0)	0.724

**Table 5:** MANOVA [37] results for the interaction test among recovery techniques (RT) and the forwarding technique (FT) for the recovery phase of GHR. * Degrees of freedom in the F statistic are 8 and 12. If there is a significant interaction (*p-*value < 0.05) in the “all metrics together” test, then interaction tests per metric need to be performed.

Number of Vehicles	Performance Metric	Wilk’s Λ	F (2,18)	*p*-Value
100	All metrics together *	0.358	1.784	0.06
	All metrics together *	0.228	5.07	0.006
	**% of packet losses**	**0.658**	**4.771**	**0.022**
150	Average delay	0.907	0.919	0.095
	**Average No. of hops**	**0.514**	**8.51**	**0.003**
	% of idle time	0.761	3.866	0.055
	All metrics together *	0.334	2.996	0.043
	**% of packet losses**	**0.706**	**3.05**	**0.041**
250	**Average delay**	**0.548**	**7.415**	**0.004**
	Average No. of hops	0.798	2.275	0.202
	**% of idle time**	**0.508**	**8.718**	**0.002**

**Table 6:** MANOVA [37] results of the testing difference in performance metrics among routing recovery techniques (RT). There is a significant difference when the *p-*value < 0.05.

Metric	Number of Density	Forwarding Factor	Wilk’s Λ	F (2,18)	*p*-Value
% of Packet Losses	100	∞ & 3	0.357	16.210	0.0001
	150	∞	0.662	4.592	0.024
	150	3	0.679	4.261	0.031
	**250**	∞	**0.894**	**1.067**	**0.365**
	250	3	0.427	12.076	0.001
Average delay	100	∞ & 3	0.204	35.17	0.0001
	150	∞ & 3	0.169	44.11	0.0001
	250	∞	0.167	44.99	0.0001
	250	3	0.65	129.924	0.0001
Average number of hops	100	∞ & 3	0.217	32.486	0.0001
	150	∞	0.198	36.398	0.0001
	150	3	0.081	63.96	0.0005
	250	∞ & 3	0.241	28.373	0.0005
% of idle time	100	∞ & 3	0.110	72.73	0.0001
	150	∞ & 3	0.219	32.05	0.0001
	250	∞	0.234	29.478	0.0001
	250	3	0.493	9.251	0.002

**Table 7:** Pairwise comparison of the performance metrics in which there is a difference among recovery techniques for the GHR protocol (i.e., *p*-value < 0.05 in Table 6). The table shows only the results for metrics and pairs of recovery values with the absence of statistically-significant differences (i.e., *p-*value ≥ 0.05).

Metric	Number of Density	Forwarding Factor	Pairwise	*p*-Value
% packet losses	150	∞	(β=3, β=0)	0.279
	150	3	(β→∞, β=0)	0.336
	250	3	(β=3, β=0)	0.562
Average number of hops	150	∞	(β=3, β=0)	0.106
% of idle time	150	3	(β=3, β=0)	0.068

## Abbreviations

AP	Access Point
CAAR	Coherent, Automatic Address Resolution
CW	Contention window
DTN	Delay-Tolerant Networks
FIFO	First In First Out
GHR	Geographical Heuristic Routing
GPS	Global Position System
GPSR	Greedy Perimeter Stateless Routing
ITS	Intelligent Transportation System
MAC	Medium Access Control
MANET	Mobile Ad hoc Network
MANOVA	Multivariate Analysis of Variance
MFR	Most Forward within R
MMMR	Multi-Metric Map-aware Routing
QoS	Quality of Service
RSU	Road Side Unit
SA	Simulated Annealing
TSRP	Tabu Search-based Routing Protocol
V2V	Vehicle-to-Vehicle
V2I	Vehicle-to-Infrastructure
VANET	Vehicular Ad hoc Network
VLOCI	VANET Location Improve
